# The biomechanics of the locust ovipositor valves: a unique digging apparatus

**DOI:** 10.1098/rsif.2021.0955

**Published:** 2022-03-16

**Authors:** Rakesh Das, Shmuel Gershon, Benny Bar-On, Maryam Tadayon, Amir Ayali, Bat-El Pinchasik

**Affiliations:** ^1^ School of Mechanical Engineering, Faculty of Engineering, Tel Aviv University, Tel Aviv 6997801, Israel; ^2^ School of Zoology, Faculty of Life Sciences and Sagol School for Neuroscience, Tel Aviv University, Tel Aviv 6997801, Israel; ^3^ Department of Mechanical Engineering, Ben-Gurion University of the Negev, Beer-Sheva 84105, Israel; ^4^ Technische Universität Dresden, B CUBE–Center for Molecular Bioengineering, Dresden 01307, Germany; ^5^ Department of Biomaterials, Germany Max-Planck-Institute of Colloids and Interfaces, Am Mühlenberg 1, 14476 Potsdam, Germany

**Keywords:** oviposition, biomechanics, digging, numerical simulations, mechanical properties

## Abstract

The female locust has a unique mechanism for digging in order to deposit its eggs deep in the ground. It uses two pairs of sclerotized valves to displace the granular matter, while extending its abdomen as it propagates underground. This ensures optimal conditions for the eggs to incubate and provides them with protection from predators. Here, the direction-dependent biomechanics of the locust's major, dorsal digging valves are quantified and analysed under forces in the physiological range and beyond, considering the hydration level as well as the females' sexual maturation state. Our findings reveal that the responses of the valves to compression forces in the digging and propagation directions change upon sexual maturation to follow their function and depend on environmental conditions. In addition, mature females, which lay eggs, have stiffer valves, up to approximately 19 times the stiffness of the pre-mature locusts. The valves are stiffer in the major working direction, corresponding to soil shuffling and compression, compared with the direction of propagation. Hydration of the valves reduces their stiffness but increases their resilience against failure. These findings provide mechanical and materials guidelines for the design of novel non-drilling burrowing tools, including three-dimensionally printed anisotropic materials based on composites

## Statement of significance

The female locust lays its eggs underground in order to protect them from predators and to provide them with optimal conditions for hatching. In order to dig into the ground, it uses two pairs of valves: the ventral pair is plugged as a wedge, while the dorsal pair performs the digging of the oviposition tunnel. We study the mechanical response of the digging valves, depending on age, hydration level and direction of operation. Our findings show that, during the course of approximately two weeks in the life of the adult female, the digging valves become up to 19 times stiffer against failure, in order to fulfil their function as diggers. While hydration reduces the stiffness, it also increases the resilience against failure and renders the valves unbreakable within the estimated physiological force range and beyond. The digging valves are consistently stiffer in the digging direction than in the perpendicular direction, indicating their form-follows-function design.

## 1. Introduction

Insects have evolved excellent biological features that promote subsurface exploration and even motion in the subterranean environment [1–4]. The relations between structure, mechanical properties and function are manifested in various successful digging mechanisms in nature [5–7]. Some of them rely on enlarged front limbs [7], while some rely on specialized body movements [4]. Several studies presented bioinspired diggers based on such digging apparatuses [8–11]. One promising example is found in the methods and structures used in insects', specifically grasshoppers', oviposition or egg laying [12–16]. Some of these methods rely on ad hoc digging apparatuses.

Egg laying is a central aspect of the reproductive biology of insects. The deposition of eggs in a carefully selected spot, in or on a carefully selected substrate, represents a major decision and a crucial act that the female insect executes to ensure the survival of its progeny. Evolution and natural selection have, therefore, acted to perfect the related mechanisms and maximize the chances of successful oviposition. One manifestation of the above is a striking diversification of the dedicated apparatuses in different insects, ensuring the extreme and intricate adaptations of the related body structures to their function and to the selected environment and substrate [13].

**Figure 1.**
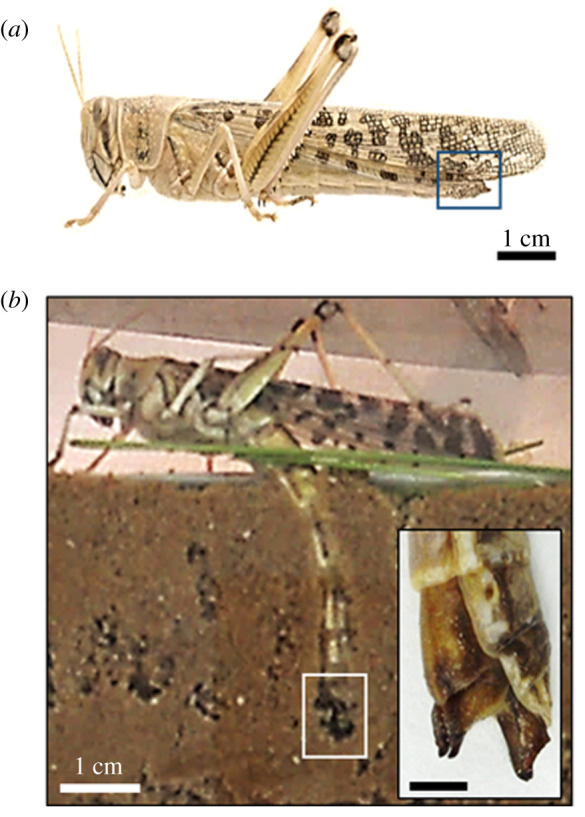
The female locust oviposition underground. (*a*) The ovipositor of the female locust at the tip of the abdomen (noted by the blue square). (*b*) A female locust during oviposition, extending its abdomen into the ground while digging. Inset: higher magnification of the two pairs of digging valves in their open state. Scale bar corresponds to 5 mm.

The ovipositor of grasshoppers and locusts is a highly specialized structure consisting of two pairs of shovel-shaped cuticular valves, a ventral and a dorsal pair, extending beyond the distal end of the female's abdomen (figure 1*a*). The valves are hinged at their bases to each other and to a prominent pair of internal apodemes, a ridge-like ingrowth of the exoskeleton, serving the large supporting muscles [17]. These structures are used to dig a deep and narrow chamber in the ground for egg burial, to manipulate the eggs and to assist in capping the egg pod with froth (figure 1*b*). During oviposition, the ovipositor valves undergo rhythmic cyclical opening and closing and retraction and protraction movements [16]. These movements are produced by the contractions of 10 pairs of muscles innervated by the terminal abdominal ganglion. Grasshoppers and locusts are rare among insects in having ovipositor valves that work by opening and closing movements rather than by valves sliding upon each other [15]. The locust ovipositor valves exert different forces needed for digging, clearing debris from the digging path and for the hyperextension of the female's abdomen during the oviposition. These tasks are divided between the valves: the ventral ones are mostly responsible for the pulling action, while the dorsal valves are used for clearing debris.

In newly hatched female locusts, the valves are visible as paired outgrowths of the posterior margins of the last abdominal segments. They enlarge and differentiate in a series of steps across the five larval stages; by the third larval instar, the valves adopt the adult ventral/dorsal orientation. In the fourth and fifth larval stages, the valves continue to enlarge with each moult, such that they extend beyond the abdomen tip at the final moult to adulthood.

The soft valves enlarge further and become densely sclerotized during an additional two to three weeks while the animal reaches sexual maturity [18–20]. How the mechanical properties of the locust ovipositor valves change with the adult stage and, specifically, during sexual maturation to facilitate digging for oviposition remains an open question.

In order to understand how the shape and mechanical response of organs serve their function in nature, specifically in arthopods [21–24], and to develop design and mechanical guidelines for bioinspired diggers [25], the quantification of biomechanical factors is essential [26–28]; namely, the physiological force range, force–deformation behaviour, maximal force before failure and the dependence of the mechanical response on direction. In addition, in the case of insect cuticles, one of the major factors influencing the mechanical properties is the hydration state [29–31].

In this study, we quantify the mechanical response of the locust ovipositor dorsal valves, depending on age (sexual maturation state), hydration and force direction. We focus on the dorsal valves since their shovel-shaped apex region plays the primary role in shuffling and compressing the soil. We explore two major force directions, corresponding to the direction of propagation and direction of burrowing, i.e. to the protraction and opening of the valves, respectively. We approximate the physiological force range in which the valves operate and discuss the mechanical stability of the valves within this range and beyond. We use compression mechanical tests to reveal the relations between an applied force and deformations of the valves, depending on hydration level. In addition, we quantify the maximal forces that the valves can withstand in the two above-mentioned directions. We quantify how the structural stiffness and maximal load-bearing force are altered with age (i.e. sexual maturation) and adapt to the mechanical needs during oviposition. Finally, we use finite-element (FE) simulations, based on three-dimensional micro-computed tomography (μCT) scans, in order to model the load distribution in the valves during digging, and we elucidate the failure mechanism of the valves in experiments. Our findings shed light on the mechanical requirements for fulfilling the function of digging underground and can inspire the development of synthetic three-dimensionally printed valves with direction-dependent improved mechanics [32–34].

## 2. Material and methods

### 2.1. Sample preparation

Locusts were obtained from our desert locust (*Schistocerca gregaria*) colony at the School of Zoology, Tel Aviv University, Tel Aviv, Israel. Ovipositor valves were dissected from females of two different groups: (i) sexually mature females (age ≥ 30 days post moult to adult, with oviposition history) and (ii) younger, pre-mature females (age 7–9 days post moult). After extraction of the valves, they were cleaned and kept in a sealed tube at room temperature (25°C). If required, the valves were preserved in a freezer (−20°C) for a maximum of 48 h. To avoid desiccation, the samples were surrounded by wet cotton and sealed with Parafilm. These preparation and preservation procedures were previously demonstrated to have no significant influence on the samples' biomechanical properties [35]. To dehydrate the samples, they were sequentially immersed in ethanol of increasing concentrations (30%, 50%, 75% and 100%).

### 2.2. Micro-computed tomography scanning-based three-dimensional model

The ovipositor valves were scanned by a μCT machine (Easytom, RX-Solutions, Chavanod, France) at an isometric voxel size of 5 µm at 125 µA and tube voltage of 80 kV, equipped with a micro-focus tube (X-ray150, RX-Solutions) and a flat panel detector (CsI scintillator). The three-dimensional model was built after the reconstruction and segmentation of three-dimensional scanned images based on the grey value using Simpleware Scan IP software (Synopsys, CA, USA). In this segmentation process, different built-in tools (median filter, island removal and flood fill) were used to reduce the noise. The segmented three-dimensional model was meshed in an FE model in the advanced meshing module of Simpleware Scan IP with tetrahedral elements (C3D4). The number of elements was 74 802, in the model of the dorsal valve tip. This meshed three-dimensional model was further used for numerical simulation. A mesh convergence study was performed to make sure that the element size has no influence on the analysis.

### 2.3. Bending cantilever experiments

**Figure 2.**
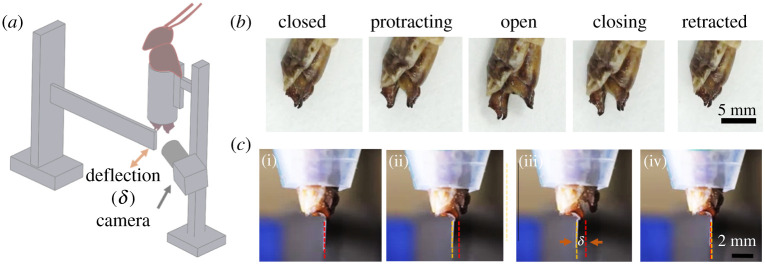
Operation and estimated physiological force range of the locust valves. (*a*) An illustration of the experimental bending beam set-up used to measure the approximated physiological force range exerted on the dorsal valves during digging. (*b*) The different states of valves during digging. (*c*) The cantilever bending experiment. The red dashed and the yellow dashed lines correspond to the original and deflected positions of the beam, respectively. *δ* is the cantilever deflection.

The experimental set-up (figure 2*a*) included a sample holder, in which the locust abdomen is fixed, while the valves are free to perform the digging motions. A steel cantilever was located so as to barely touch the dorsal valves. We took advantage of the well-established fact that the oviposition motor programme can be activated by releasing the local control system from descending inhibition, i.e. by transection of the ventral nerve commissures caudal to the abdominal ganglia [12]. The digging movements of the valves induced displacement of the beam, and this displacement was imaged using a camera (Panasonic DC-S1 with Sigma 70 mm *f*/2.8 DG Macro lens). The generated force is considered a point load, as the diameter of the dorsal tip is considerably smaller than the deflecting edge of the beam. ImageJ freeware was used to analyse the images and extract the cantilever deflection (*δ*). The force was extracted from the cantilever deflection via *p* = 3*δEI*/*L*^3^ [36], where *L* = 5.2 cm is the cantilever length, *I* = *WT*^3^/12 is the cantilever moment of inertia with *W* = 0.27 cm and *T* = 0.1 cm and *E* = 180 GPa is the Young modulus of the cantilever's material.

### 2.4. Tip-loading mechanical tests

The shafts of the valves were embedded in epoxy glue onto a screw-head, exposing only the apex region. The screw was mounted on a customized grip of a mechanical testing system (TA Instruments, USA) in order to make the base of the samples immobile when the tip of the samples touched the compression plate. A preload of 0.2 N was applied through the compression plate on the tip. A constant displacement rate of 0.005 mm s^−1^ was applied on the tip, with a total displacement of 0.7 mm. The corresponding force was recorded by the load cell of the system. The two reference directions of the applied compressive force were chosen based on the functioning of the valves during the digging. The force–displacement curves (*F*–*d*) were obtained from this experiment, in which the initial slope represents the elastic stiffness and the compressive breaking force was calculated from the maximum of these curves.

### 2.5. Scanning electron microscopy

The fractured specimens of the ovipositor valves were imaged using a scanning electron microscope (SEM) (GeminiSEM300; Zeiss, Oberkochen, Germany). The fractured specimens were obtained after a compression mechanical test (see above) in two reference force directions. Before scanning, the samples were sputter-coated using a Polaron sputter coater (model SC7640, Au-Pd target; Polaron England).

### 2.6. Finite-element simulation for the mechanical behaviour of the valves

The three-dimensional meshed models of the dorsal and ventral valves pair were exported to the Abaqus-FEA package (v. 6.14; ABAQUS Inc., CA, USA) for analysis through FE simulation. The average material properties determined from nanoindentation (see electronic supplementary material, Supporting information, *Nanoindentation tests* and figure S2) were applied in these models. During the simulation, the valves are considered to be linear elastic and isotropic. The boundary conditions for the compression mechanical test were applied. The base of the valves was fixed (all translational and rotational degrees of freedom were constrained), and the force determined from the compression mechanical experiment was applied at the tip of the valves. The resulting principal stress distributions were analysed.

## 3. Results

### 3.1. Physiological force measures of the valve

To quantify the physiological force range that the female locust's digging apparatus operates in, we performed a bending cantilever experiment (figure 2).

We activated the oviposition motor programme by transection of the ventral nerve commissures caudal to the abdominal ganglia [12], thus inducing movements of the ovipositor valves resembling those demonstrated during natural digging behaviour (see §2.3).

Figure 2*a* illustrates the experimental set-up, in which the locust's abdomen is fixed while the valves are induced to perform the digging motions. As noted above, during digging, the valves go through cyclic opening and closing (figure 2*b*). Initially, the valves are in their closed/retracted state. The ovipositor then protracts, opens and closes and retracts in a complete cycle [37]. A full cycle, under the conditions used in this experiment, corresponds to approximately 3–4 s. This is in agreement with the time span of a full digging cycle underground (see electronic supplementary material, video V1).

A representative image sequence of the bending beam experiment is shown in figure 2*c* (see electronic supplementary material, video V2). During the opening movements of the valves, the cantilever experiences deflection (figure 2*c*(ii,iii)), and the force is calculated (see electronic supplementary material, Materials and methods). Any abdominal movements beyond those of the valves are restricted to cancelling the potential influence on the beam deflection. This calculation yielded a force corresponding to 0.85 ± 0.15 N (*n* = 10) in the direction of opening (perpendicular to the propagation). Since the experiment is conducted in air, the actual forces exerted by the intact female locust may differ during real operation underground (owing to resistance or feedback from the granular matter). Nevertheless, this measurement and calculation provide an estimate of the physiological forces for later discussion and correspond to the maximal digging force applied by the female.

### 3.2. Mechanical response of the locust valves to tip loadings

**Figure 3.**
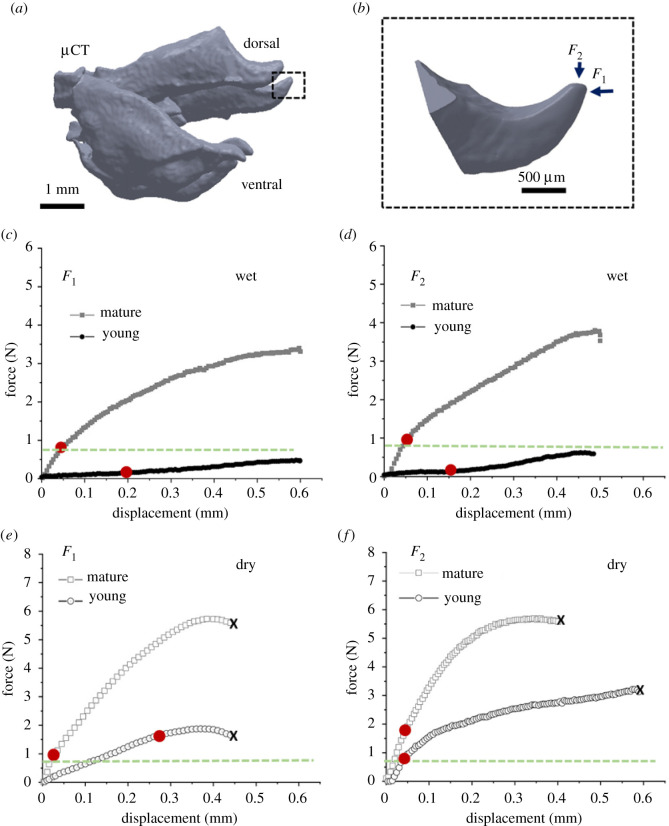
Mechanical response of the locust valves under uniaxial compression. (*a*) µCT reconstruction of the entire dorsal and ventral valves and (*b*) a higher magnification of the dorsal valve's tip. Two major force axes are noted: in the direction of propagation, *F*_1_, and in the direction of burrowing, *F*_2_. (*c*,*d*) Representative force–displacement curves of mature (grey) and younger pre-mature (black) hydrated (wet) valves under forces *F*_1_ and *F*_2_. (*e*,*f*) Representative force–displacement curves of mature (grey squares) and young (black circles) dehydrated (dry) valves under forces *F*_1_ and *F*_2_. The end of the linear range, which represents the elastic stiffness response, is marked with red dots on each of the curves in the hydrated and dehydrated conditions, respectively. The x indicates the point of failure (occurs only in dry state). The green dashed lines represent the maximum of the physiological force range.

We quantified the mechanical response of the locust dorsal valves to normal and lateral tip loadings, which correspond to its propagation and digging actions, respectively (figure 3).

Figure 3*a* shows a µCT scan reconstruction of the entire dorsal and ventral valves. Figure 3*b* shows a higher magnification of the dorsal valve's tip under load in the two major axes: the direction of propagation, *F*_1_, and the direction of burrowing, *F*_2_.

Representative force–displacement curves are shown in figure 3*c*–*f*. We quantify the response both in the estimated physiological range of up to approximately 0.85 N, marked by green dashed lines in the plots, and further until failure of the valves. We compare valves of mature (grey) and younger pre-mature (black) locusts and quantify the role of hydration in their mechanical stability in the two force directions. Four main points are evident: (i) valves of mature locusts are significantly stiffer than those of young locusts, (ii) valves are stiffer in the *F*_2_ direction than in the *F*_1_ direction, (iii) hydrated (wet) valves are less stiff but more resilient to failure, in both the physiological operation range and beyond, and (iv) hydration influences mostly the mechanical properties, namely stiffness, of the younger locusts compared with the mature ones.

**Figure 4.**
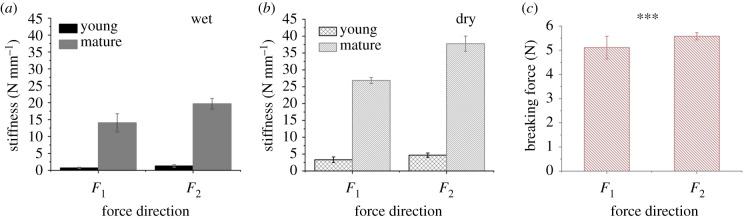
The age, hydration and direction-dependent mechanical response of the locust valves. The structural stiffness of the dorsal valve's tip of young and adult locusts in (*a*) hydrated (wet) and (*b*) dehydrated (dry) conditions. (*c*) Breaking force of the valves in dehydrated conditions (***p ≤ 0.01, Student's *t*-test).

Figure 4 summarizes the mechanical response of the dorsal valve's tip depending on age and hydration level (figure 4*a,b*) and presents the breaking force of dehydrated valves in both directions (figure 4*c*).

In the physiological range, the structural stiffness of the dorsal valves of a mature locust in the wet state in the *F*_1_ direction corresponds to 14.08 ± 2.64 N mm^−1^ (*n* = 10), which is almost 19 times the stiffness of the valves of the pre-mature locusts, with 0.75 ± 0.19 N mm^−1^ (*n* = 10) (figure 4*a*). The stiffness was calculated from the initial linear slope of the force–displacement curve. Interestingly, wet valves did not break under compression, even for displacements of 0.6 mm, corresponding to forces of up to approximately 4 N in the mature locusts and 0.6 N in the young locusts. However, an irreversible residual deformation was visible in all hydrated samples (electronic supplementary material, figure S1). In force direction *F*_2_, the stiffness of the wet mature locust valves in the physiological range corresponds to 19.69 ± 1.60 N mm^−1^ (*n* = 10), which is approximately 140% the stiffness in the wet state in the *F*_1_ direction of 14.08 ± 2.64 N mm^−1^ (*n* = 10). In addition, in the *F*_2_ direction, the wet valves of the mature locusts are significantly stiffer than the valves of the wet young ones, with 19.69 ± 1.60 N mm^−1^ and 1.30 ± 0.35 N mm^−1^ for the mature and young locusts, respectively (figure 4*a*).

While the hydration level of the valves is unknown in living locusts, we observe a strong dependence of the mechanical properties on the hydration state. The stiffness is, as expected, higher for dry valves, corresponding to approximately twice the stiffness of the wet ones for both force directions in mature locusts (figure 4*a*,*b*). Namely, 26.83 ± 0.89 N mm^−1^ compared with 14.08 ± 2.64 N mm^−1^ in the *F*_1_ direction for mature locusts, for dry and wet valves, respectively, and stiffnesses of 37.75 ± 2.27 N mm^−1^ compared with 19.69 ± 1.60 N mm^−1^ in the *F*_2_ direction, for dry and wet conditions, respectively. Also in the dehydrated valves, the structural stiffness in the *F*_2_ direction is higher than that in the *F*_1_ direction, with 37.75 ± 2.27 N mm^−1^ (*n* = 10) compared with 26.83 N mm^−1^, respectively.

It is well established that insects' cuticles lose water during sclerotization [38,39]. With our results, we are able to quantify the influence of hydration between young and mature locusts. For example, for a young locust, the structural stiffness in the *F*_1_ direction increases from 0.75 ± 0.19 N mm^−1^ (wet) to 3.32 ± 0.83 N mm^−1^ (dry). This corresponds to an increase of approximately 340% in stiffness. However, for a mature locust, the increase in stiffness is only 90%, from 14.08 ± 2.64 N mm^−1^ to 26.83 ± 0.89 N mm^−1^. Most likely, this is because of the lower water content in the more sclerotized areas of the organ [20].

While in the estimated physiological force range we did not observe failure of the valves, in either the young or the mature valves, in wet or dry states, we observed failure of the valves in higher compression forces, within the range of 0.6 mm, corresponding to the maximal displacement in the experiment. None of the valves failed in the wet conditions. However, the valves of the mature locust failed when they were dehydrated under compression forces of 5.58 ± 0.15 N in the *F*_2_ direction compared with 5.11 ± 0.47 N in the *F*_1_ direction. While these values are far from the physiological conditions, they still maintain the trend of increased mechanical stability in the direction of digging (*F*_2_).

### 3.3. Mechanical failure

**Figure 5.**
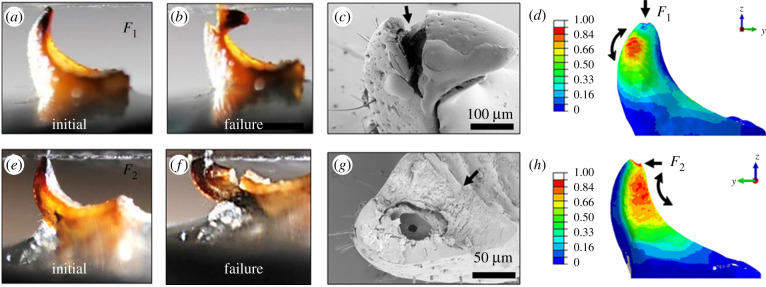
Analysis of the mechanical failure of the locust valves: experiments and simulations. Optical micrographs of (*a*) the valve in the initial state and (*b*) after failure under force in direction *F*_1_. (*c*) Scanning electron micrograph of the valves after their mechanical failure; the black arrow indicates the direction of fracture. (*d*) Simulated normalized maximal principal stress in the valves under force *F*_1_. Equivalent images and simulations for force direction *F*_2_ are depicted in (*e*–*h*).

We now focus on the structural position and the mechanism of the mechanical failure of the dorsal valves in their dry state, under forces in the propagation (*F*_1_) and digging (*F*_2_) directions (figure 5). Figure 5*a*–*d* and *e*–*h* show optical micrographs of the valves in the initial state and after failure, SEM images of broken valves and FE simulations of the maximal principal stress distribution in the dorsal valves in *F*_1_ and *F*_2_ directions, respectively. The fracture topography implies a brittle material [40], agreeing with the force–displacement curves of the dry valves (figure 3*e*,*f*). Therefore, the criterion for failure corresponds to the maximal tension developing in the valves under external forces [41].

In order to understand the failure mechanism of the valves, the stresses developing under external loads were modelled. To this end, we mapped the maximal principal stress distribution in the dorsal valves under applied forces in the *F*_1_ and *F*_2_ directions using FE simulations. The normalized stresses are shown in figure 5*d*,*h*, respectively.

For the simulations, we used the µCT scans of the dorsal valves (figure 3*b*). The base of the valves was fixed with the full encastre condition, and forces *F*_1_ and *F*_2_ were applied at the tip of the valve with a magnitude of 5 N, corresponding to the breaking force obtained from the compression tests. The forces were applied in the directions corresponding to the compression tests: *F*_1_ was applied by defining pressure at the valve's tip in the direction of propagation, and *F*_2_ was applied by defining surface traction at the tip in the direction of soil removal. The material was assumed to be linear elastic and isotropic. The elastic modulus was defined as *E* = 5 GPa, which is the average elastic modulus of mature dry dorsal valves, measured by nanoindentation (see electronic supplementary material, figure S2). Poisson's ratio was defined to be 0.38. [6]

Figure 5*d* presents the convex side of the valves, corresponding to the internal side of the digging system (figure 2*b*), while figure 5*h* presents the concave side, corresponding to the external side in the digging system. The black curved arrows indicate the direction of the tensile principal stress component at the location of its maximal value. During compression under *F*_1_, the failure of the valve occurs at the convex region (the spine of the valve), as shown in figure 5*d*. We find remarkable agreement between the area of maximal tensile stress in the simulation and the point of failure in the experiment (figure 5*b*,*c*). We exclude concentrated stresses at load application areas. Similarly, the area of the maximal tensile stress, under *F*_2_ in the simulation, agrees with the compression experiment (figure 5*e*). In this case, the failure starts from the concave surface, where the maximal tensile stress develops.

## 4. Discussion

The female locust has a unique way of digging in granular material, in which the ground is being compressed to the sides of the burrow rather then removed, and for which the digging valves are remarkably adapted. During underground burrowing, the digging valves of the female locust are subjected to forces of approximately 0.85 N in the direction of digging. The stiffness of the valves increases almost 19-fold until the locust reaches sexual maturation, within two weeks. This remarkable difference shows that the valves' mechanical stability increases significantly in preparation for their function as digging tools during oviposition.

Considering the force in the physiological operation conditions, the valves are entirely resilient to failure when in their hydrated conditions. Dehydration increases the stiffness of the valves but also renders the valves more prone to fracture under load. Nevertheless, this occurs in forces more than five times higher in magnitude than the characteristic forces in the physiological conditions. Regardless of the hydration level, the valves are stiffer in the digging direction than in the propagation direction, implying that their design meets the criteria for functioning as resilient digging apparatuses. Dehydrated valves are brittle and fail mechanically owing to the development of local tensile forces. Because of the structural gradient of the valves, the failure occurs close to the tip of the valves.

Overall, the valves' structural gradient, together with the reinforcement of their stiffness upon the locusts' sexual maturation and the dependence on hydration, may set clear guidelines for the design of synthetic diggers. First, the structural gradient ensures that the failure happens closer to the tip, which may allow the organ to operate even if fractured. Second, the stiffness of the valves should be adjusted to the designated granular matter and environmental conditions. Third, water, which functions as a plasticizer, increases the resilience of the digger against fracture and failure. It is, therefore, a trade-off between the stiffness and compliance of the material, in order to offer both functionality and resilience.

To mimic cuticular materials and specifically the digging organs of insects, three-dimensional printing using composite materials is an attractive direction. Specifically, it provides an ability to alter the mechanical properties of the printed material in specific regions and in specific directions. Considering that such three-dimensionally printed composites are very often brittle [42], we provide simulations of the maximal tension developed in the organ during loads in both the propagation and digging directions. These provide guidelines for the design and possible material re-enforcement where the maximal tension develops in order to improve the resilience of bioinspired systems against failure [43].

## 5. Conclusion

The mechanical response of the locust valves corresponds to their biological function and changes dramatically throughout the female locust's life cycle. The stiffness of the valves of mature locusts is almost 20 times higher than that of younger locusts, and the valves' stiffness is higher in the direction of digging than in the direction of propagation. The hydration of the valves makes them more resilient against failure, showing no breakage in the physiological force range of digging and beyond. The stiffness of the valves of the sexually mature locusts, however, is less affected by dehydration than that in the younger locusts, probably because of a lower water content in the mature adult valves. Finally, FE simulations elucidate why the valves break in specific places, toward the tip, in both force directions. The tensile stress distribution indicates the area in which the tip is most likely to break, as observed in mechanical compression experiments. This is a striking example of the form-follows-function principle in natural structures that can inspire technological innovations and bioinspired mechanical tools for robotics.

## Data Availability

Additional data are available in the electronic supplementary material [44].
